# Cross-Atlantic differences in reading the same breast screening cases

**DOI:** 10.1186/bcr2979

**Published:** 2011-11-04

**Authors:** Y Chen, AG Gale, MG Evanoff, U Zakir

**Affiliations:** 1 Loughborough University, Loughborough, UK; 2 American Board of Radiology, Tucson, AZ, USA

## Introduction

American breast screening radiologists typically read a lower annual volume and recall more cases than their UK counterparts. This study investigated what happens when experienced breast screening radiologists from both countries examined the same FFDM case set, albeit using different resolution displays.

## Methods

Sixteen experienced American breast screening radiologists interpreted 40 difficult FFDM cases containing various mammographic features, excluding small calcifications, using dual 20^2 ^DICOM calibrated monitors. For comparison purposes, the anonymous data were used of 16 experienced UK breast radiologists who had read the same cases as part of the PERFORMS scheme using clinical mammographic workstations.

## Results

The 16 American radiologists were split into two groups of low volume (<5,000 cases p.a.) and high volume (≥5,000 cases p.a.) and performances were compared. There was no significant differences (*t *= 0.23, *P *> 0.05). Consequently their performance data were combined and compared with those of the 16 UK radiologists. There was no significant difference between the two groups in correct recall decisions (UK, 97.1%; USA, 92.9%; *t *= 0.042, *P *> 0.05) although there were significant differences in correct return to screening decisions (UK, 88.9%; USA, 80%; *t *= 0.089, *P *< 0.05) and the number of malignancies detected (UK, 98.7%; USA, 93%; *t *= 0.049, *P *< 0.05).

## Conclusion

The use of lower resolution monitors (approximately half that of a mammographic workstation) by the American group was offset by their experience (>15 years) such that even very experienced but low-volume readers performed well. Whilst the UK group overall performed better on these cases, the American group still recalled more, reflecting their real-life screening criteria.

